# ^13^C-labeled biochemical probes for the study of cancer metabolism with dynamic nuclear polarization-enhanced magnetic resonance imaging

**DOI:** 10.1186/s40170-015-0136-2

**Published:** 2015-09-14

**Authors:** Lucia Salamanca-Cardona, Kayvan R. Keshari

**Affiliations:** Department of Radiology and Molecular Pharmacology and Chemistry Program, Memorial Sloan Kettering Cancer Center (MSKCC), 1275 York Avenue, New York, NY 10065 USA

**Keywords:** Hyperpolarized, Nuclear magnetic resonance, Nuclear magnetic imaging, Dynamic nuclear polarization

## Abstract

In recent years, advances in metabolic imaging have become dependable tools for the diagnosis and treatment assessment in cancer. Dynamic nuclear polarization (DNP) has recently emerged as a promising technology in hyperpolarized (HP) magnetic resonance imaging (MRI) and has reached clinical relevance with the successful visualization of [1-^13^C] pyruvate as a molecular imaging probe in human prostate cancer. This review focuses on introducing representative compounds relevant to metabolism that are characteristic of cancer tissue: aerobic glycolysis and pyruvate metabolism, glutamine addiction and glutamine/glutamate metabolism, and the redox state and ascorbate/dehydroascorbate metabolism. In addition, a brief introduction of probes that can be used to trace necrosis, pH changes, and other pathways relevant to cancer is presented to demonstrate the potential that HP MRI has to revolutionize the use of molecular imaging for diagnosis and assessment of treatments in cancer.

## Review

### Introduction

Since the hallmark discovery of the Warburg effect in cancer cells in the 1920s, it has been widely accepted that the metabolic properties of cancer cells are vastly different from those of normal cells [1]. Starting from the observation that many cancerous (neoplastic) cells have higher rates of glucose utilization and lactate production, the development of tools and methods to correlate specific cellular metabolic processes to different types of cancer cells has received increased research focus [2, 3]. Several imaging techniques are currently in use for this purpose, including radiography, scintigraphy, positron emission tomography (PET), single-photon emission computed tomography (SPECT), and magnetic resonance (MR) [4, 5].

For more than 30 years, MR has been a revolutionary diagnostic tool, used in a wide range of settings from the central nervous system to cardiomyopathies and cancers. MR imaging (MRI) can outline molecular and cellular processes with high spatial resolution. Typically, MRI of body tissues is achieved via contrast visualization of the protons (^1^H) of water, which are present in high abundance in living systems. This can be extended to MR spectroscopy (MRS), which can further differentiate between less abundant, carbon-bearing, biological metabolites in vivo utilizing ^1^Hs of these compounds [6, 7]. However, despite its usefulness in imaging whole body tissues, ^1^H MRS has low spectral resolution and poor sensitivity for these less abundant metabolites. In addition, ^13^C MRS is increasingly difficult, in comparison to ^1^H MRS, in that both the gyromagnetic ratio (approximately 25 % of ^1^H) and natural abundance (1.1 % of ^1^H) are significantly lower, making the detection of carbon-bearing compounds difficult [8, 9]. The low spectral resolution of ^1^H MRS for metabolites can be addressed by using ^13^C-enriched compounds, and with this direct ^13^C MRS, metabolic processes can be traced, utilizing enriched tags on specific carbons in a given metabolite [10]. While enrichment of molecules in ^13^C can also moderately address the sensitivity limitation of MRS, recent work in hyperpolarization (HP) provides a means of dramatically increasing sensitivity and enhancing signals, well beyond that of the equilibrium state obtained via MRS. [11, 12]. The focus of this review will be the introduction of this approach in the setting of cancer metabolism, delineating probes of interest, which have been applied to study metabolic processes in vivo.

### Obtaining a hyperpolarized probe

In MR, a desired target is placed in a magnetic field where the nuclear spins of molecules are aligned with or against the direction of the magnetic field. The nuclear spins have thus different energies, and an MR signal is detected upon relaxation of nuclear spins of higher energy. At thermal equilibrium, the number of spins aligned with the magnetic field nearly equals the number of spins opposing the direction of the magnetic field. Thus, at thermal equilibrium, spin polarization is in the order of >0.0005 % resulting in a limited signal. Signal increases on the order of 100,000-fold can be achieved by hyperpolarizing the system via the redistribution of the spin population levels found at equilibrium [10, 13]. There are several techniques that have been used to achieve hyperpolarization of various nuclei: spin-exchange optical pumping of ^3^He and ^129^Xe, parahydrogen-induced polarization (PHIP), and dissolution dynamic nuclear polarization (DNP) [11, 14, 15]. Both PHIP and DNP techniques can polarize biologically relevant nuclei like ^13^C and ^15^N, although there is a wider range of molecules that can be targeted for hyperpolarization using dissolution DNP [14, 16–18].

The goal of DNP is the transfer of polarization from highly polarized unpaired electron spins to the nuclear spins of a desired target compound. This is achieved by applying an external magnetic field to a free-radical agent in order to polarize electron spins, followed by saturating the electron spin resonance via microwave irradiation in order to obtain polarization transfer. The free-radical agent is generally a stable organic compound that is compatible with aqueous buffers, which are used as solvent in order to obtain a homogeneous distribution of the radical [13]. Nearly 100 % of the electrons on the free-radical agent are polarized when the free-radical/solvent mixture is subjected to high magnetic fields (≥3.3 T) followed by rapid freezing to 1 K using liquid helium in order to obtain a sample frozen to an amorphous state, which is necessary for retention and transfer of polarization [18]. For biological applications, after transfer of electron spin polarization to the nuclei of interest has occurred, the preparation must exist in solution, which can be achieved utilizing a dissolution process in which the solid sample is rapidly melted via injection of a hot solvent, typically a biologically compatible buffer, into the frozen sample [13]. The dissolution process results in a liquid sample at room temperature, while still preserving the enhanced polarization obtained by the microwave irradiation of the frozen sample [8]. Additionally, the use of chelating agents (e.g., EDTA) with the solvent to eliminate trace metals and more recently the use of gadolinium (Gd) chelates with the DNP sample have been used to further enhance and retain polarization in the liquid sample, albeit with caution over potential toxic effects when applied in vivo and the potential for loss of hyperpolarization due to *T*_1_ shortening [11, 19, 20]. More in-depth exploration of the technical aspects of probe development has been previously reviewed [8, 11].

### Considerations in probe selection and current research

The usefulness of a molecule for hyperpolarized MRS is dependent on the polarization lifetime of the nucleus of interest, and this property is determined by the spin-lattice relaxation constant (*T*_1_) [21]. Dipolar coupling, the magnetic field range, and molecular size can also affect the *T*_1_ of a given nucleus. In general, high magnetic fields and large molecular weights decrease the *T*_1_. Dipole-dipole coupling of ^13^C with ^1^H is common in biologically relevant molecules, and it shortens relaxation times; therefore, carbon atoms directly bound to ^1^H are generally not useful as probes for HP. For example, all carbons present in glucose (an important substrate in cancer cells) have relaxation times shorter than 2 s [22]. On the other hand, carbonyl carbons of biologically relevant molecules generally have *T*_1_’s above 20 s even at high magnetic fields like [1-^13^C] pyruvic acid, which has relaxation times of 67, 48, and 44 s at 3, 11.7, and 14.1 T, respectively [23–25]. Even carbons that are less oxidized than carbonyls, like the hemi-ketal in [2-^13^C] fructose have *T*_1_’s one order of magnitude higher than glucose carbons. Short spin-lattice relaxation times can sometimes be increased by deuterium enrichment of the sample. With this technique, protons that are directly bound to carbons are exchanged for deuterium atoms which results in the reduction of dipole-dipole relaxation, further preserving the hyperpolarized state [26]. This has resulted in increased *T*_1_’s of ^13^C nuclei in molecules such as glucose (*T*_1_ increased from 2 s to 10–14 s), providing the possibility of utilizing them in future metabolic studies [27–29]. Despite the effect of deuterium enrichment, research efforts have largely focused on developing carbonyl-bearing molecules as molecular imaging probes. The focus of this review is to introduce representative compounds relevant to metabolism that are characteristic of cancer tissue and have been applied in the work of multiple groups: aerobic glycolysis, glutamine addiction, and the redox state.

#### Pyruvate and aerobic glycolysis

**Fig. 1:**
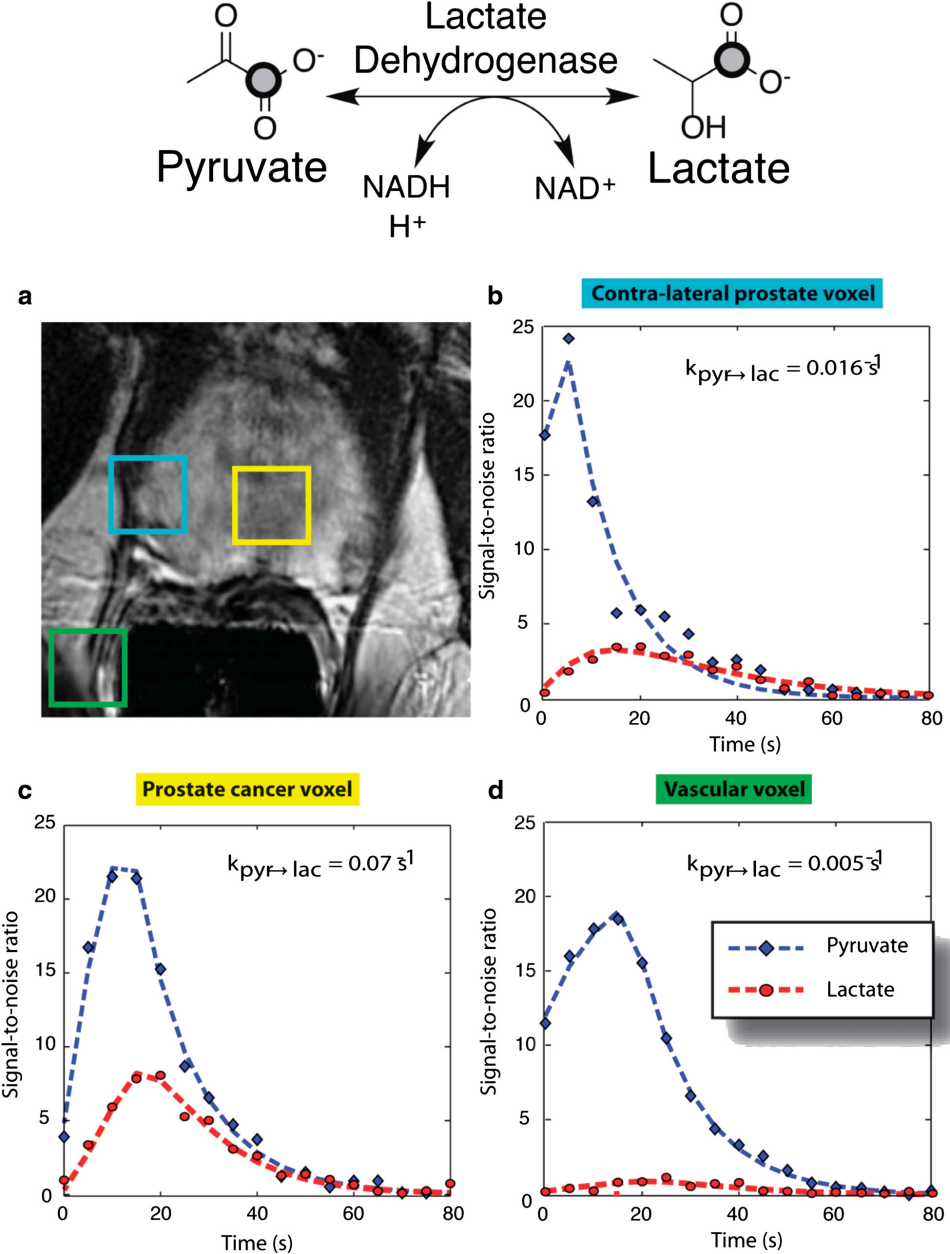
Flux of hyperpolarized [1-^13^C] pyruvate to [1-^13^C] lactate in prostate regions. **a** MR image from patient with prostate cancer showing regions of cancerous tissue and surrounding normal tissue. **b**–**d** Localized dynamic hyperpolarized [1-^13^C]pyruvate and [1-^13^C]lactate spectral from voxels overlapping the contralateral region of prostate (*turquoise*), a region of prostate cancer (*yellow*), and a vessel outside the prostate (*green*). Adapted with permission from ref. [43]

Of particular interest to cancer metabolism is the increased conversion of glucose to lactate as a result of modulated aerobic glycolysis. This process, also known as the Warburg effect, is characteristic of many tumors with altered metabolism where pyruvate generated from glucose metabolism via glycolysis is preferentially converted to lactate by lactate dehydrogenase (LDH) as opposed to entering the tricarboxylic acid cycle [1]. With this phenotype, cancer cells show a preference for lactate fermentation even in the presence of oxygen, thus bypassing oxidative respiration for ATP generation. Because of this, pyruvate has been the preferred probe for HP MRS research since it is an intermediate metabolite in pathways characteristic of aberrant metabolism in cancer cells, including increased lactate production as a result of aerobic glycolysis where detection of HP pyruvate-derived lactate can be used as a marker for cancer and response to treatment [30, 31] as well as an intermediate in amino acid metabolism (e.g., interconversion to alanine via transamination with glutamate) (Fig. 1). In addition, as mentioned before, carbonyl carbons in pyruvate have long relaxation times where even the methyl carbon can have *T*_1_’s above 50 s after deuterium enrichment [32]. The interconversion of pyruvate to lactate has been exploited for MRI by using [1-^13^C] pyruvate and detecting the accumulation of increased lactate in cancerous tissue as compared to surrounding benign tissue. Increased conversion of pyruvate to lactate and alanine has been demonstrated to precede MYC-driven tumorigenesis by using HP [1-^13^C] pyruvate in murine models [33]. Furthermore, in the same study, a decrease in the flux of alanine was observed at the tumor stage while a decrease in lactate conversion was indicative of tumor regression [33]. In transgenic adenocarcinoma of mouse prostate (TRAMP) models, in vivo studies using HP [1-^13^C] pyruvate demonstrated that hyperpolarized pyruvate and its metabolic products can be used non-invasively and with high specificity to obtain a profile of the histologic grade of prostate cancers [34]. In vivo imaging following hyperpolarized pyruvate has also been used to evaluate the role of glutaminase and LDH in human lymphoma models [35] as well as to elucidate metabolism of pyruvate in breast cancer [36] and renal cell carcinoma with treatment [30, 37].

Early work that utilized HP pyruvate to assess the response of tumors to treatment was conducted in mice xenografted with EL-4 lymphoma cells and treated with etoposide, a topoisomerase inhibitor that causes rapid cell death [38, 39]. In this study, tumor necrosis was correlated to a decrease in the flux of hyperpolarized lactate which was suggested to be due to a decrease in NAD+ and NADH in the intracellular pool as well as loss of LDH function. More recently, HP [1-^13^C] pyruvate has been used as a biomarker to evaluate early response to radiation therapy in glioma tumors by observing a decrease in hyperpolarized lactate suggested to be a result of changes in tumor perfusion which can be detected between 24 and 96 h following treatment [40, 41]. HP [1-^13^C] pyruvate has also been used to detect early response to temozolomide (TMZ) treatment on human glioblastoma rat models [42]. The study successfully showed for the first time detection of response to TMZ therapy 1 day after TMZ administration. The continued reports on using HP pyruvate as an imaging probe for assessing treatment response indicate the potential of the compound to become a standard in the field. Moreover, these studies demonstrate the usefulness of HP [1-^13^C] pyruvate as a tool for early assessment of therapy response, which can improve treatment selection at the clinical level. Pyruvate has also been validated as a metabolic imaging marker for use in humans [43]. In a two-phase study, patients with biopsy-proven prostate cancer of various histological grades were injected with HP [1-^13^C] pyruvate. In the first phase, a maximum dose level was determined to establish pharmacological safety of the HP probe while still injecting enough pyruvate to allow visualization. This addressed one of the major challenges faced in translating HP MRI to clinical applications: the potential toxicity of compounds that must be injected into patients. In the second phase, metabolism of pyruvate was visualized in real time and differences in the ratio of [1-^13^C] lactate to [1-^13^C] pyruvate between identified cancerous regions and normal tissue regions were successfully observed (Fig. 1a–d). [1-^13^C] lactate in regions that did not contain tumor was not detected, confirming previous biopsy and preclinical studies that demonstrated low flux of [1-^13^C] pyruvate to lactate and low concentrations of lactate in benign prostate tissues [44, 45]. Preliminary results indicated the possibility of detecting previously unobserved cancerous regions by HP [1-^13^C] pyruvate, later confirmed to be Gleason 4+3 cancer by biopsy, though further investigation into the relationship between grade and metabolism in prostate cancer patients is needed. While there are challenges associated with translation to clinical use for HP [1-^13^C] pyruvate, the first in human study demonstrated the feasibility of hyperpolarization technology as a safe diagnostic tool and provides the potential for utilizing this approach in preclinical models with direct translation to the clinic.

#### Glutamine metabolism

**Fig. 2:**
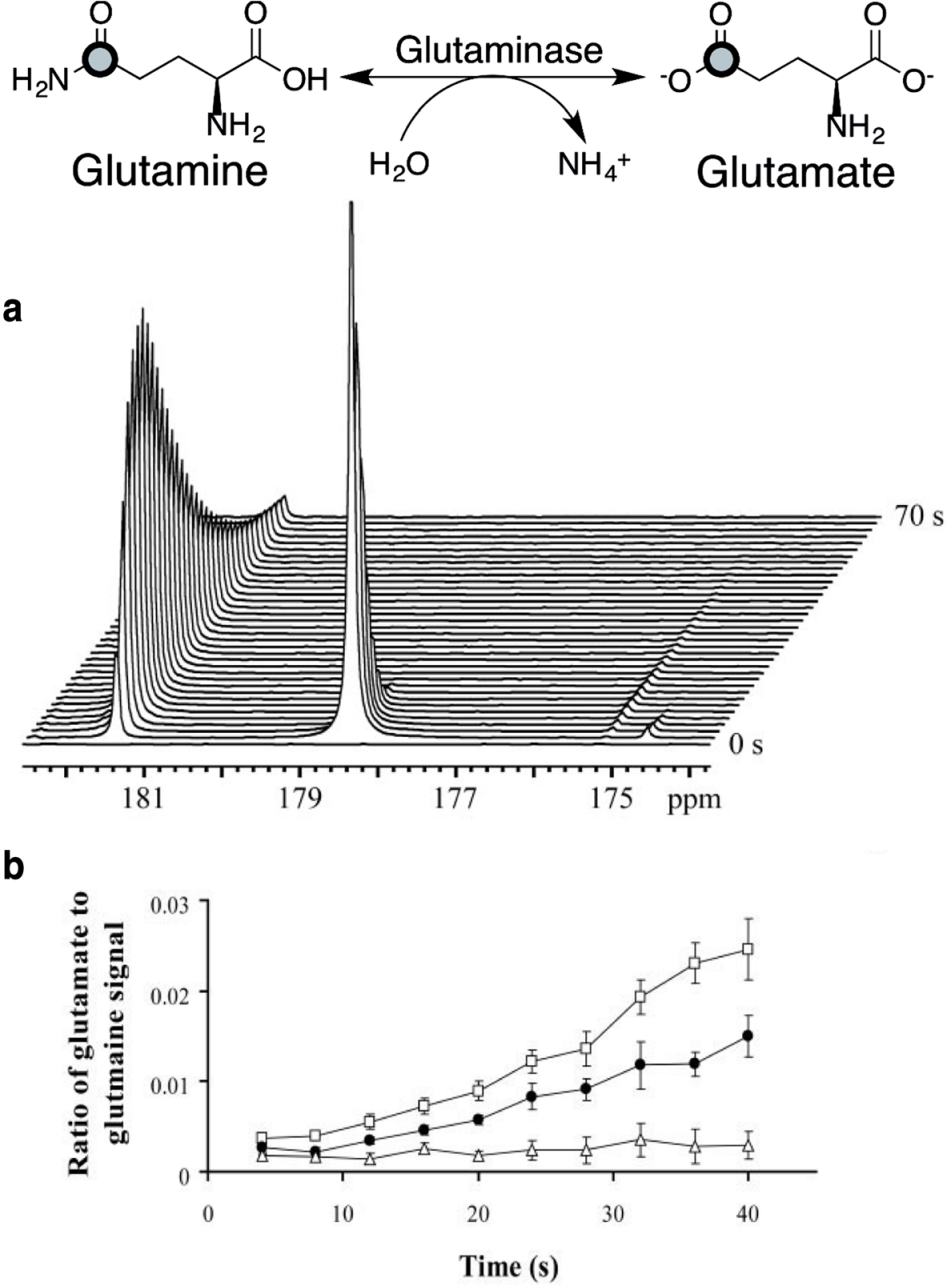
Metabolism of [5-^13^C] glutamine to [5-^13^C] glutamate. **a** Time-dependent spectral data following conversion of [5-^13^C] glutamine to [5-^13^C] glutamate. The signals are from ^13^C-enriched [5-^13^C]glutamate at 181.5 ppm and [5-^13^C]glutamine at 178.5 ppm and from natural abundance ^13^C label in [1-^13^C]glutamate at 175.2 ppm and [1-^13^C]glutamine at 174.7 ppm. **b** Plot of the ratio of the signal intensities of [5-^13^C]glutamate/[5-^13^C]glutamine showing the ratio in hepatoma cells (*shaded circle*), cell lysate (*square*), and control (*triangle*). These results demonstrated that hepatoma cancer cells convert glutamine to glutamine at a higher rate than normal cells. Adapted with permission from ref. [49]

Glutamine is an amino acid that plays an important cellular role as nitrogen donor in the form of an amide group for purine and pyrimidine biosynthesis, leaving a glutamate molecule in the process although glutamine can also be converted to glutamate by glutaminase in a reaction independent of nucleotide biosynthesis. Glutamate is the primary nitrogen donor for the biosynthesis of non-essential amino acids. Transaminases catalyze the transfer of the amine group from glutamate to α-ketoacids to synthesize alanine, aspartate (precursor for asparagine), serine (precursor for glycine and cysteine), ornithine (precursor for arginine), and proline which is derived from the glutamate carbon backbone. Glutamine is considered a non-essential amino acid as it can be recycled from glutamate and ammonia in a reaction catalyzed by glutamine synthetase; however, some cancer cells show increase consumption of glutamine and are unable to grow in the absence of exogenous glutamine [46, 47]. This metabolic characteristic of cells to require exogenous glutamine for growth has been termed “glutamine addiction” and has generated extensive research interest as an indicator of development of cancerous tissues [48]. In particular to the field of HP MRI, the conversion rate of glutamine to glutamate (Fig. 2) was explored in hepatocellular carcinoma (HCC) using a [5-^13^C] glutamine probe (Fig. 2) [49]. Using the ratio between [5-^13^C] glutamine and [5-^13^C] glutamate, it was demonstrated that HCC cells convert glutamine at a higher rate than normal cells supporting the notion of glutamine addiction. One important aspect of this study was the choice of [5-^13^C] glutamine as a probe as opposed to [1-^13^C] glutamine, which has a longer *T*_1_ (16.1 vs. 24.6 s at 9.4 T) [49, 50]. [5-^13^C] glutamine was selected because the chemical shift change obtained from [1-^13^C] in glutamine and glutamate is far too small, which could prevent proper identification and quantification of the peaks. This highlights the importance of understanding not only the target compound to be hyperpolarized but also the metabolic products to be detected and their resulting spectra in MR. This is further emphasized with studies that demonstrate the usefulness of [1-^13^C] glutamine as a source for [1-^13^C] glutamate in order to follow the metabolism of α-ketoglutarate to observe the metabolic state of the TCA cycle in transformed cells [51]. Furthermore, [1-^13^C] α-ketoglutarate has been hyperpolarized and used to visualize other metabolic events involving [1-^13^C] glutamate such as mutations in IDH1 expression in glioma tumors and pathways dependent on hypoxia-inducible factor (HIF) [51–53]. More recently, [5-^13^C] glutamine has been used to visualize the metabolism of liver cancer in vivo and in vitro, as well as the treatment response of prostate cancer cells in vitro [54]. Based on the promise of glutamine as a biomarker for cancer diagnosis and treatment response, extending the spin-lattice relaxation time of the [5-^13^C] glutamine has been researched and successfully accomplished. The facile synthesis of [5-^13^C-4-^2^H_2_] glutamine has been reported, and its study showed that by relying on the effect of deuterium enrichment to lessen dipolar coupling effects, the *T*_1_ of [5-^13^C] glutamine could be increased from approximately 15 to 30 s [55]. Visualization of real-time conversion of glutamine to glutamate in SF188 cells was achieved using this probe, demonstrating the promise of [5-^13^C-4-^2^H_2_] glutamine as a probe for molecular imaging of metabolic events in real time. Further investigation of this probe applied to in vivo preclinical models will lay the foundation for its clinical translational potential in the future.

#### Dehydroascorbate as a redox sensor

**Fig. 3:**
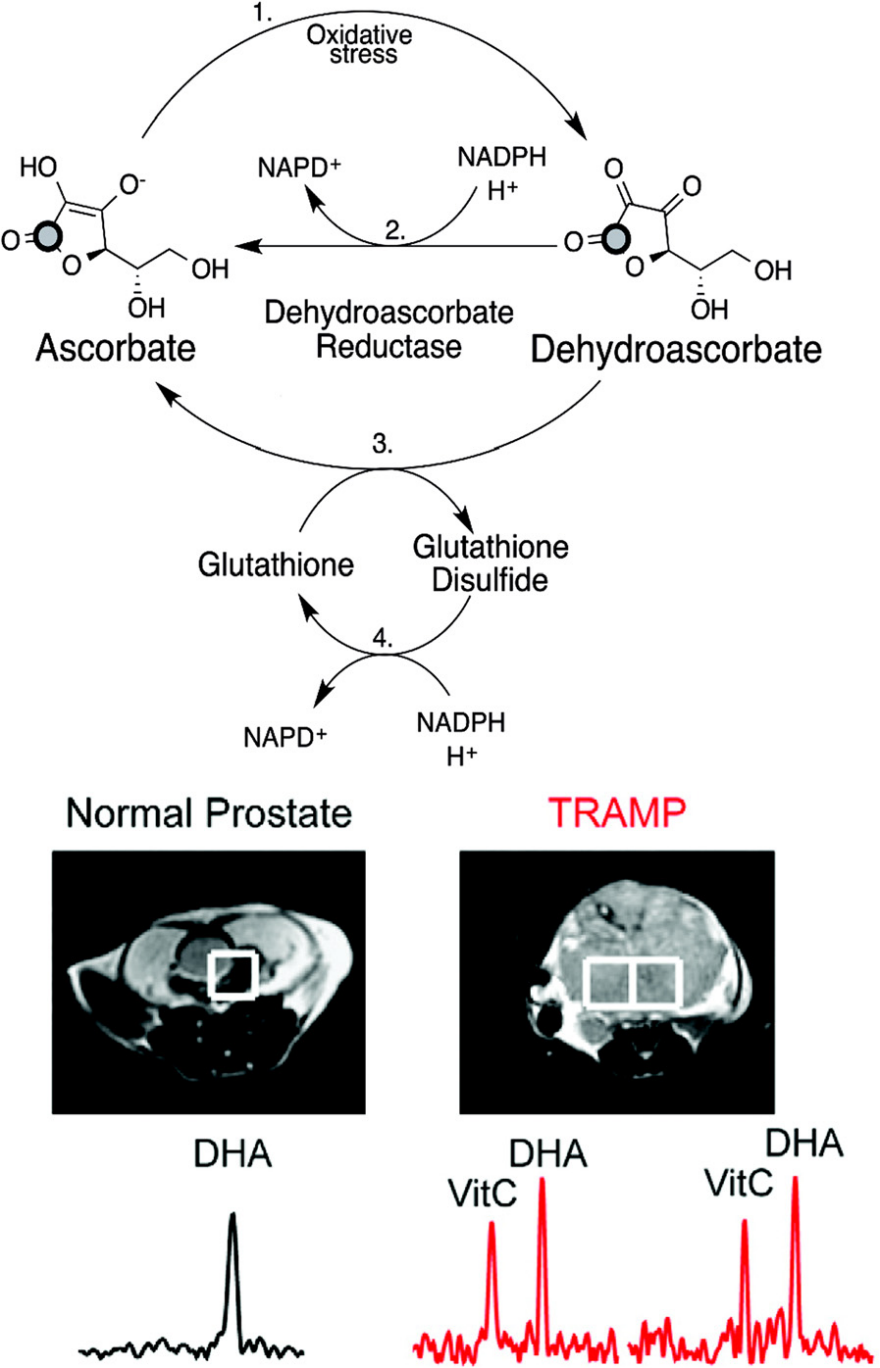
Determination of redox state by imaging of HP [1-^13^C] ascorbate (VitC) and [1-^13^C] dehydroascorbate (DHA). Oxidative stress caused by ROS (*1*.) can be alleviated by oxidation of ascorbate to DHA (*2*.), and recycling of DHA to ascorbate can occur indirectly with oxidation of glutathione (*3*.) or directly with oxidation of NADH (*4*.). The ratio of [ascorbate] to [DHA] has been successfully used in mice models as a biomarker to determine pH in vivo. Adapted with permission from ref. [62]

Reactive oxygen species (ROS) like the hydroxyl radical, superoxide, and hydrogen peroxide have been shown to cause DNA damage and can lead to mutations that transform normal cells into cancerous cells [56]. The reduction/oxidation (redox) state, which is dependent on the balance between oxidizing equivalents like ROS and reducing cofactors, can provide insight into the physiological condition of the cell with respect to potential cancer transformations. Furthermore, the presence of ROS in tissue has been implicated to be a factor in developing resistance to radiation therapies [57]. During oxidative stress (i.e., when there is an increase in ROS), redox homeostasis is maintained by the action of antioxidant compounds, such as ascorbate (or vitamin C, VitC), which can scavenge for ROS and reduce the compounds to rid the cells of damaging agents [58]. In this process, ascorbate that is available to cells in high concentrations can be oxidized to dehydroascorbate (DHA) while reducing ROS. DHA can then be transported into the cell where DHA is reduced back to ascorbate resulting in a process of recycling ascorbate and DHA (Fig. 3) [59]. In this sense, the ratio of DHA to ascorbate can be used as a molecular marker to investigate the redox state and thus the physiological state of tissues. Additionally, conversion of DHA to ascorbate can be enzymatically catalyzed in an NADPH-dependent manner or via oxidation of glutathione (GSH) to glutathione sulfide (GSSG); thus, visualization of ascorbate/DHA metabolism offers a method for probing in vivo metabolism of NADPH as well as determination of GSSG to GSH ratio, both of which have been implicated to be indicators of oxidative stress in the cells, particularly for neurodegenerative, cardiovascular, and cancer diseases [60–62]. Hyperpolarized [1-^13^C] DHA was successfully used in murine models to detect increased reducing capacity in prostate cancer with the purpose of developing a non-invasive, early diagnostic tool for improving selection of treatment therapies [62, 63]. DHA demonstrates a relatively long *T*_1_ at clinically relevant field strengths (>50 s at 3 T) and adequate chemical shift separation between it and its metabolic product ascorbate (*δ* = 3.8 ppm). Increased reduction of HP [1-^13^C] DHA to ascorbate was observed in tumor tissue compared to normal tissue as well as other metabolic organs (Fig. 3). This was additionally demonstrated in lymphoma cells, further supporting the potential for using DHA as a probe in living systems [64]. A following study validated these results, and the correlation between increased DHA reduction and glutathione was established in vivo, thus showing the utility of [1-^13^C] DHA as a molecular imaging probe to detect events that go beyond the direct metabolism of DHA [63]. Notwithstanding the potential of HP DHA as a diagnostic probe, the toxicity of DHA remains to be validated. Earlier studies on mammalian cells showed DHA toxicity starting at 10 mM, while a study carried on rats demonstrated neurological effects of DHA starting at injections of 50 mg/kg [65, 66]. However, as outlined above, successful use of DHA injections in rats and mice for hyperpolarization has been demonstrated without reported side effects on the animals. More research is needed to determine the parameters regarding the toxicity of DHA in larger animal models using pure formulations to assess its potential for clinical trials. Further work in DHA could demonstrate its applicability for the study of ROS and redox changes in model systems.

### Other metabolic imaging probes

While the three probes discussed earlier are the most well studied in metabolic events that are characteristic of cancer cells in general, other molecules have been evaluated in their potential to be used as biomarkers. Hyperpolarized bicarbonate (H^13^CO_3_^−^) has been successfully used to determine the pH in extracellular matrix of lymphoma tumors in mice, and a correlation between acidic environments and cancer was established [67]. The relaxation times for bicarbonate compounds at 3 T are between 34 and 50 s, which is enough time to detect the rapid conversion of H^13^CO_3_^−^ and ^13^CO_2_ catalyzed by carbonic anhydrase [23]. The attractive feature of this probe is based on how ubiquitous acidic extracellular environments are to a wide variety of diseases; thus, HP bicarbonate has the potential for clinical translation beyond cancer research, though extensive work will be necessary to generate a preparation which will result in an adequate dose for the clinic [68, 69]. More recently, the potential of α-ketoisocaproate (KIC) as a molecular probe for in vivo detection of branched chain amino acid transaminase (BCAT) has been explored. BCAT catalyzes the conversion of KIC to leucine, and its expression has been suggested to correlate to genetic characterization of certain tumors. In a pilot study, HP α-keto-[1-^13^C]-isocaproate was shown to have a *T*_1_ of 100 s so its metabolism can be sensitively traced for over a minute after injection [70]. In the same study, metabolism of HP [1-^13^C] KIC to [1-^13^C] leucine by BCAT was observed in murine lymphoma tumor tissue but was absent in rat mammary adenocarcinoma with a correlation between BCAT expression and [1-^13^C] leucine signal detection [70]. Additionally, in the same models, [1-^13^C] pyruvate conversion to [1-^13^C] lactate and [1-^13^C] alanine was detected in both types of tumors. These findings show the promise of using [1-^13^C] KIC as a discriminative probe in addition to pyruvate in order to diagnose different types of cancer [71, 72]. Furthermore, the correlation between BCAT expression and [1-^13^C] leucine detection was also shown in rat brain tissue, confirming the usefulness of HP [1-^13^C] KIC in assessing BCAT activity in vivo [73]. Choline is another compound that has been evaluated as a molecular imaging probe since elevated choline and choline-derived metabolites have been correlated by ^1^H-MRS imaging to cancer in the brain, breast, colon, cervix, and prostate [74–76]. Despite its potential as a global marker for cancer because of the long *T*_1_ relaxation times that can be achieved with deuterium and ^15^N enrichment [77, 78], HP applications of ^13^C enriched choline are limited due to the small change in chemical shifts of choline and choline-derived metabolites as well as its potential toxicity [16, 79, 80]. It has been shown that choline toxicity occurs at doses of 53 mg/kg in mice, although a recent study successfully detected HP ^13^C choline in vivo without adverse effects in rats at doses of 50 mg/kg by using atropine to prevent cholinergic intoxication [81, 82] though metabolic products have been difficult to visualize in vivo. As mentioned earlier, the usefulness of glucose as a probe is limited due to the short relaxation times of all the carbons present in the molecule and although the *T*_1_’s can be increased through deuterium enrichment, the lifetime of the probe remains a hurdle for clinical applications [27, 28]. Thus, fructose (a pentose analog of glucose) has been successfully used as an alternative to probe glycolytic pathways [83] in TRAMP models where differences in HP [2-^13^C] fructose uptake and metabolism was visualized in tumor regions compared to surrounding normal tissues. Like choline, the limiting factor in the usefulness of [2-^13^C] fructose for in vivo studies is in small chemical shifts between the metabolite and its phosphorylated product. Finally, tumor necrosis can be used as a measure of treatment response, particularly early necrosis. HP [1,4-,^13^C] malate has been visualized in lymphoma mice models after injection of HP [1,4-^13^C] fumarate [84]. In normal cells, fumarate has a slow rate of transport into the mitochondria; however, in necrotic cells where the mitochondrial membrane is degraded, fumarase has access to the HP fumarate and its ubiquitous cofactor, water, thus facilitating rapid conversion to malate. Preliminary studies have shown the potential for its use in animal models though further work is required to determine the necessary density of necrotic cells for detection and the timings required for adequate visualization in patients.

## Conclusions

**Fig. 4:**
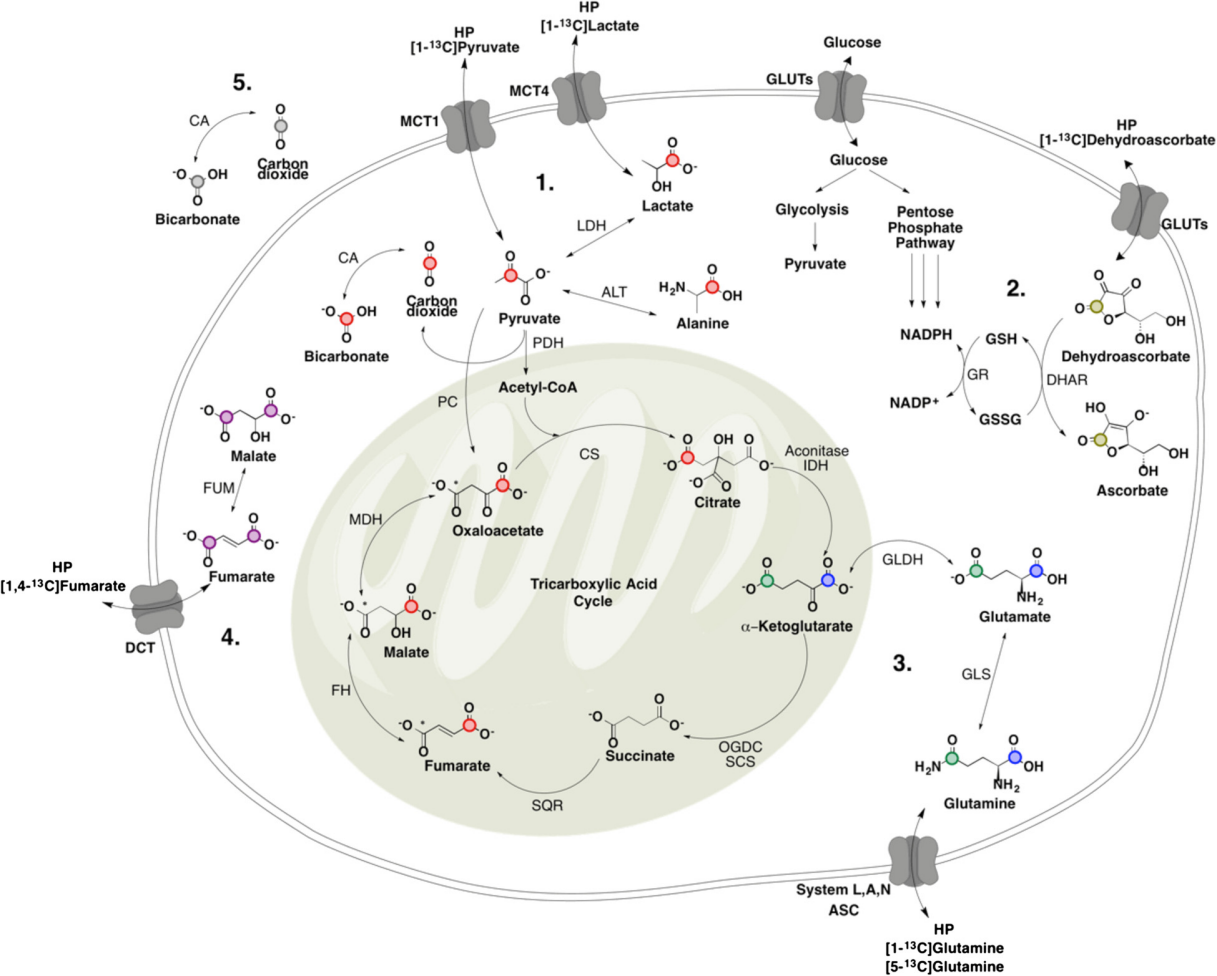
Metabolic pathways with compounds that can be used as molecular imaging probes for HP MRI. A wide variety of metabolic pathways have already been visualized or have the potential to be visualized using hyperpolarization technology that can be applied to different pathological states of the cell including cardiovascular disease and a large variety of cancers. *1*. Metabolism of C1 (*red dots*) in pyruvate. The *asterisks* on selected compounds represent enrichment of ^13^C in the second pass of pyruvate in TCA cycle. *2*. Metabolism of C1 (*brown dots*) in DHA using a pool of NADPH derived from the pentose phosphate pathway. *3*. Metabolism of C1 (*blue dots*) and C5 (*green dots*) of glutamine. *4*. Metabolism of C1 and C4 (*purple dots*) of fumarate unrelated to TCA metabolites. *5*. Metabolism of extracellular bicarbonate (*gray dots*). *MTC1* monocarboxylate transporter 1, *MTC4* monocarboxylate transporter 4, *System ASC* amino acid transporter, *GLUTs* glucose transporters, *DCT* dicarboxylate transporter, *DHAR* dehydroascorbate reductase, *GR* glutathione reductase, *GSH* glutathione, *GSSG* glutathione disulfide, *LDH* lactate dehydrogenase, *ALT* alanine transaminase, *CA* carbonic anhydrase, *PC* pyruvate carboxylase, *PDH* pyruvate dehydrogenase, *CS* citrate synthase, *GLS* glutaminase, *GLDH* glutamate dehydrogenase, *IDH* isocitrate dehydrogenase, *OGDC* oxoglutarate dehydrogenase complex, *SCS* succinyl CoA synthetase, *SQR* succinate dehydrogenase, *FH* fumarate hydratase, *MDH* malate dehydrogenase, *FUM* fumarase. Cofactors have been omitted for brevity

The application of hyperpolarized ^13^C imaging has been extensively investigated in preclinical models, and the successful demonstration of HP [1-^13^C] pyruvate in patients with prostate cancer has validated the potential of HP MRI as a safe diagnostic and treatment assessment tool. Application of other probes beyond pyruvate is still in its infancy, particularly because of the need to further study the currently developed models under conditions that are relevant to a clinical setting (i.e., lower magnetic fields) as well as to study the necessary parameters (probe toxicity dose limits, safety limits for rapid injection) to withstand the necessary hurdles to translation. Nevertheless, these vast research findings are promising and indicate an eventual translation to humans. Furthermore, there is a large variety of biologically relevant molecules that have the potential to be hyperpolarized (Fig. 4), and molecular imaging of metabolic events in real time using not only one single probe but a combination of relevant probes could become an invaluable tool in elucidating so far undiscovered metabolic and proteomic interactions that play a role in cancer development and treatment. This gives HP MRI the great potential to revolutionize current molecular imaging technologies.

## Abbreviations

ALT: alanine transaminase
BCAT: branched chain amino acid transaminase
DHA: dehydroascorbate
DNP: dynamic nuclear polarization
EDTA: ethylenediaminetetraacetic acid
GSH: glutathione
GSSG: glutathione sulfide
HCC: hepatocellular carcinoma
HIF: hypoxia-inducible factor
HP: hyperpolarized/hyperpolarization
IDH: isocitrate dehydrogenase
KIC: ketoisocaproate
LDH: lactate dehydrogenase
MR: magnetic resonance
MRI: Magnetic resonance imaging
MRS: magnetic resonance spectroscopy
NAD(H): nicotinamide adenine dinucleotide
NADP(H): nicotinamide adenine dinucleotide phosphate
PET: positron emission tomography
ROS: reactive oxygen species
SPECT: single-photon emission computed tomography
TRAMP: transgenic adenocarcinoma of mouse prostate
